# Supplementary material for: A Mouse Model to Assess STAT3 and STAT5A/B Combined Inhibition in Health and Disease Conditions
Source: Cancers (Basel). 2019 Aug 22;11(9):1226. doi: 10.3390/cancers11091226 (PMC6770775; doi:10.3390/cancers11091226)
Supplement: Supplementary file 1 [file cancers-11-01226-s001.zip › cancers-578563-SI.pdf]

Supplementary Materials

A Mouse Model to Assess STAT3 and STAT5A/B Combined Inhibition in Health and Disease Conditions

Herwig P. Moll, Julian Mohrherr, Leander Blaa, Monica Musteanu, Patricia Stiedl, Beatrice Grabner, Katalin Zboray, Margit König, Dagmar Stoiber, Thomas Rülicke Sabine Strehl, Robert Eferl and Emilio Casanova

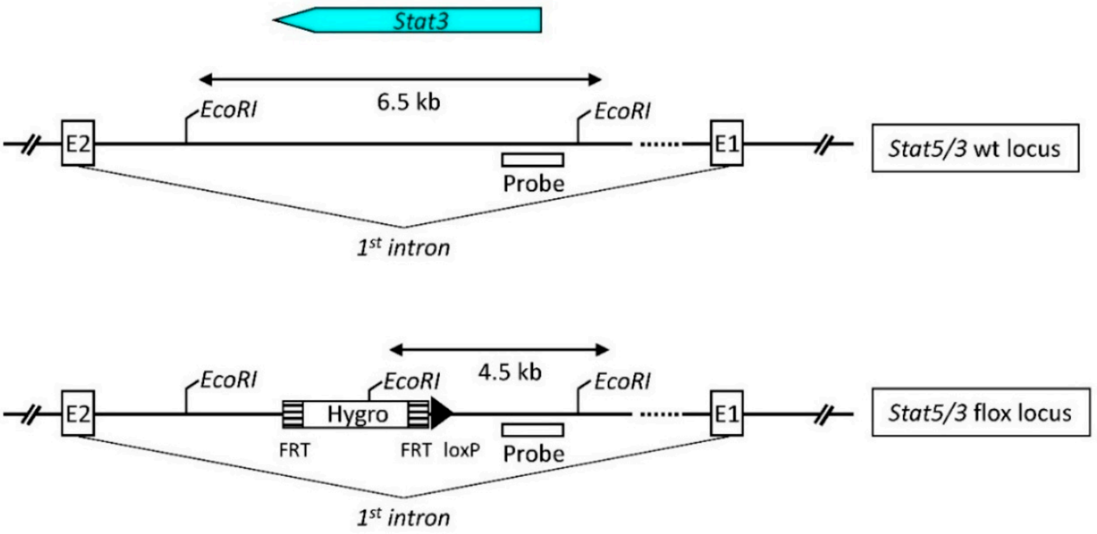

Figure S1. Schematic description of the Southern blot strategy presented in Figure 1B. Detailed representation of wt *Stat3* locus including the Southern blot probe used in Figure 1B and of the *Stat3* targeted locus containing the hygromycin cassette placed between exons 1 and 2 of *Stat3*. *EcoRI* sites used for diagnostic purposes in the Southern blot, as well as expected fragments sizes upon genomic DNA digestion with *EcoRI* are indicated.

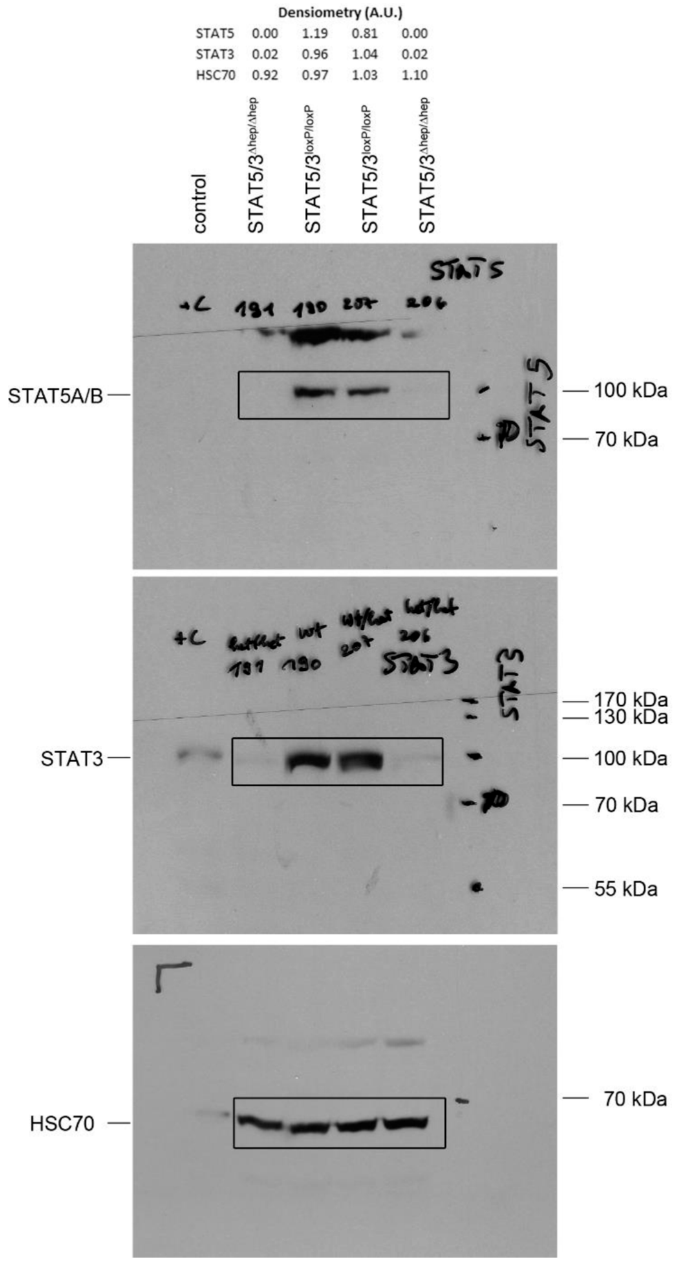

Figure S2. Densitometric quantification and uncropped Western blots related to the figure 3C.

© 2019 by the authors. Licensee MDPI, Basel, Switzerland. This article is an open access article distributed under the terms and conditions of the Creative Commons Attribution (CC BY) license (http://creativecommons.org/licenses/by/4.0/).
